# A novel marker based on necroptosis-related long non-coding RNA for forecasting prognostic in patients with clear cell renal cell carcinoma

**DOI:** 10.3389/fgene.2022.948254

**Published:** 2022-09-21

**Authors:** Jinxing Lv, Qinghui Xu, Guoqing Wu, Jian Hou, Guang Yang, Cheng Tang, Genyi Qu, Yong Xu

**Affiliations:** ^1^ Department of Urology, Zhuzhou Central Hospital, Zhuzhou, China; ^2^ Department of Urology, Dehua Hospital Affiliated to Huaqiao University, Quanzhou, China; ^3^ Department of Urology, Urology Research Institute, The First Affiliated Hospital, Fujian Medical University, Fuzhou, China; ^4^ Division of Urology, Department of Surgery, The University of Hongkong-ShenZhen Ospital, ShenZhen, China

**Keywords:** signature, long noncoding RNA, necroptosis, prognosis, clear cell renal cell carcinoma

## Abstract

**Background:** The incidence of clear cell renal cell carcinoma (ccRCC) is high and has increased gradually in recent years. At present, due to the lack of effective prognostic indicators, the prognosis of ccRCC patients is greatly affected.Necroptosis is a type of cell death, and along with cell necrosis is considered a new cancer treatment strategy. The aim of this study was to construct a new marker for predicting the prognosis of ccRCC patients based on long non-coding RNA (nrlncRNAs) associated with necroptosis.

**Methods:** RNA sequence data and clinical information of ccRCC patients from the Cancer Genome Atlas database (TCGA) were downloaded. NrlncRNA was identified by Pearson correlation study. The differentially expressed nrlncRNA and nrlncRNA pairs were identified by univariate Cox regression and Lasso-Cox regression. Finally, a Kaplan-Meier survival study, Cox regression, clinicopathological features correlation study, and receiver operating characteristic (ROC) spectrum were used to evaluate the prediction ability of 25-nrlncrnas for markers. In addition, correlations between the risk values and sensitivity to tumor-infiltrating immune cells, immune checkpoint inhibitors, and targeted drugs were also investigated.

**Results:** In the current research, a novel marker of 25-nrlncRNAs pairs was developed to improve prognostic prediction in patients with ccRCC. Compared with clinicopathological features, nrlncRNAs had a higher diagnostic validity for markers, with the 1-year, 3-years, and 5-years operating characteristic regions being 0.902, 0.835, and 0.856, respectively, and compared with the stage of 0.868, an increase of 0.034. Cox regression and stratified survival studies showed that this marker could be an independent predictor of ccRCC patients. In addition, patients with different risk scores had significant differences in tumor-infiltrating immune cells, immune checkpoint, and semi-inhibitory concentration of targeted drugs. The feature could be used to evaluate the clinical efficacy of immunotherapy and targeted drug therapy.

**Conclusion:** 25-nrlncRNAs pair markers may help to evaluate the prognosis and molecular characteristics of ccRCC patients, which improve treatment methods and can be more used in clinical practice.

## Introduction

CcRCC is one of the most common fatal malignant tumors of the urinary system, comprising a proportion of 3%–5% of all new cancers annually. It is second only to prostate cancer as well as bladder cancer of the urinary system (Aiello and Kang, 2019). ccRCC is the most common renal cell carcinoma, in the proportion of 80% of cases (Miller et al., 2019). In spite of the emergence of various novel targeted drugs and treatment strategies, the prevalence and mortality of ccRCC continue to increase year by year. (Escudier et al., 2019) In addition, ccRCC is resistant to both radiotherapy and chemotherapy (Makhov et al., 2018). Surgery is the main efficient therapy for local ccRCC, but its efficacy for advanced ccRCC is limited. Metastasis has been reported in approximately 30% of patients with ccRCC at the time of first diagnosis (Zheng and Yang, 2017). Therefore, the construction of effective prognostic indicators is of great significance for the clinical treatment of ccRCC patients. Necroptosis is a caspase-independent cell necrosis, a novel kind of apoptosis mediated *via* receptor-interacting protein kinase 1/3 (RIPK1/RIPK3) and through the mixed lineage kinase domain-like (MLKL) (Declercq et al., 2009). An increasing quantity of studies have indicated that necroptosis exerts an important function in tumors. Its role in regulating tumorigenesis and cancer progression is a double-edged sword (Gong et al., 2019). On one hand, RIPK3, as a vital mediator of the necroptosis pathway, is down-regulated in various cancers and inhibits the growth and metastasis of tumor cells (Wang et al., 2020; Tan et al., 2021). This evidence suggests that necroptosis plays an active role in inhibiting tumor progression.

In addition, studies have shown that tumors can induce necrosis of microvascular (Strilic et al., 2016; Chen et al., 2021a). On the other hand, the key MLKL is up-regulated in some cancers, which is associated with tumor hyper-aggressive behavior and immunosuppressive microenvironment (Ando et al., 2020; Yamauchi et al., 2021). Furthermore, studies have shown that tumors induce microvascular endothelial cells and promote the invasion and metastasis of cancer cells (Strilic et al., 2016; Chen et al., 2021a).

LncRNA is a protein-free coding RNA with more than 200 nucleotides in length, which is involved in special functions, such as mRNA splicing, transcriptional regulation, and post-transcriptional regulation of mRNA (Zuo et al., 2021). Studies have confirmed that LncRNAs are closely related to tumor genesis, transformation, and immunity, and can become the new potential prognostic biomarkers for tumor patients due to their excellent molecular stability (Denaro et al., 2019; Statello et al., 2021). Therefore, further elucidation of the relationship between nrlncRNAs and ccRCC is crucial for discovering new targets for drugs of ccRCC and improving the prognosis of patients.

At present, many studies have focused on the relationship between lncRNA expression standards and the prognosis of malignant tumors. Several studies have reported the prediction of prognosis of tumor patients (Hou et al., 2022) by lncRNA marker construction. (Hou et al., 2022). Recently, more and more studies have concentrated on the part of lncRNA expression in prognostic of ccRCC. Tang et al. (2021) established an immunity-associated lncRNA marker to forecast prognosis of renal clear cell carcinoma. Yu et al. (2021a) established an m6a-related lncRNA marker to forecast prognosis of ccRCC. Lei et al. (2021b) established an autophagy-associated lncRNA marker to forecast the prognosis of ccRCC. At the same time, there are few studies on predicting the prognosis of patients with malignant tumors by lncRNA markers associated with necroptosis. Among them, Wang and Liu. (2021), Chen et al. (2022a), and Lu et al. (2022) respectively constructed necroptosis-associated lncRNA markers to forecast prognostic of gastric cancer, breast cancer, and lung cancer. But there is currently no research on necroptosis-associated lncRNAs in ccRCC.

In this paper, we use necroptosis-associated lncRNAs for the first time to establish a prognosis marker to assess the prognostic of ccRCC patients. We construct a prognosis marker according to express standards of lncRNA pairs as illustrated by Hong et al. (2020) as well as independent of lncRNA expression criteria. In the paper, ccRCC transcription data can be downloaded from TCGA, and nrLncRNAs are mined by Pearson relevant study. Secondly, a 25-nrLncNA pair signature is constructed to predict the survival of ccRCC patients by univariate study and LASSO regression study. In addition, signature exploration based on the 25-nrLncRNA pairs’ signature is crucial to provide strong theoretical evidence for the application of immunotherapy and targeted drug therapy in ccRCC patients.

## Materials and methods

### Data acquisition

**FIGURE 1:**
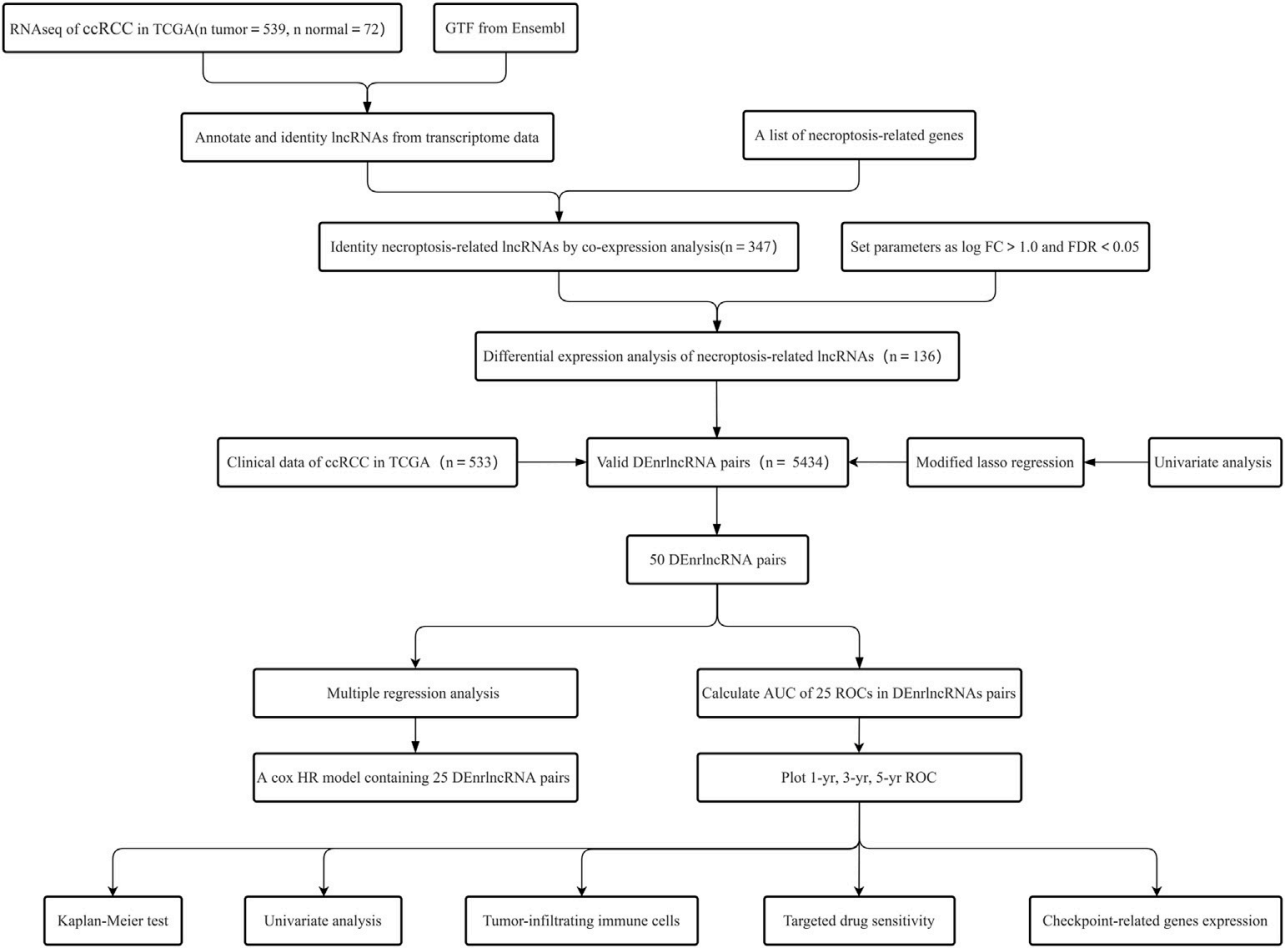
The flowchart for this article.

The detailed process of our study is illustrated in Figure 1. Transcriptome sequencing data with FPKM layout and clinic information with XML layout of 539 ccRCC patients could be obtained from the TCGA database (as of 30 March 2022) for subsequent data analysis (Tomczak et al., 2015). The FPKM information could be curated and noted using the Perl program (version Strawberry-Perl-5.30.0.1) (Nie et al., 2021) and then accessed through Ensembl Human Genome Browser (version 26) classified them into protein-coding genes and LcnRNAs (Yates et al., 2020), and screened out 533 cases with complete follow-up information for subsequent study.

The clinical characteristics of the study population are indicated in Supplementary Table S1. As the materials were sourced from the TCGA database, the TCGA-approved publication specification was strictly followed without authorization from the ethics committee.

### Identification of nrlncRNAs in ccRCC

Based on the necroptosis gene group M24779. gmt and searching published studies (Zhao et al., 2021), all sixty-seven necroptosis-associated genes could be collected (Supplementary Table S2 **)**. Pearson correlation study evaluated the correlation between death-related gene expression and lncRNAs. NrlncRNAs were selected according to correlation coefficients >0.6 and *p* < 0.001.

### Identification of differentially expressed nrlncRNAs in ccRCC

We used the “limma” package in R software to verify differentially expressed nrLncRNAs (DEnrlncRNAs) between tumors as well as adjacent ordinary tissues, *p* < 0.05 and |logFC|>1.0 were set as cutoff criteria (Ritchie et al., 2015). DEnrlncRNAs were visualized using a heat map package and volcano package.

### Establishment of differentially expressed nrlncRNAs pairs

We performed several pairing cycles analysis of these DEnrlncRNAs to define DEnrlncRNA pairs. We obtained a matrice of 0 or 1, for DEnrlncRNA pairs A|B, 0 indicates that specimen A expressed below B, and 1 means that the expression of B is lower than A in this sample. If the nrlncRNA pair has a value of 0 or 1 of all persons, expression standards of both nrlncRNAs are the same in all people, then it is not necessary to pair and build a prediction model. Thus, stable DEnrlncRNA pairs with a stability score of 20%–80% will be obtained for further study, because the 20%–80% range was used in other previous studies (Sun et al., 2021; Tang et al., 2021).

### Development of nrlncRNA pairs prognostic signature

After DElnrlncRNA pairs were obtained, univariate Cox regression was performed for each pair of DEnrlncRNA, and nrlncRNA pairs with *p* < 0.05 were screened. Then, the LASSO regression was assumed linear, we perform LASSO regression to avert over-fitting as well as obtain proper variables. Then, multivariate Cox regression was used to construct survival prediction markers. A risk score was obtained for each sample according to the prognostic marker equation constructed by multivariate Cox regression. The risk score = $\sum_{i=1}^{n}\beta iSi$ , where n represents all nrlncRNA pairs contained in the marker, where *β* represented the coefficient of nlncRNA pair i and S represented the expression of nlncRNA pair i. Risk score for every patient could be obtained for further analysis. The results of the Cox study were visualized by investigators as well as living packages in R software.

### Evaluation of the nrlncRNA pairs prognostic signature

The sensitivity and specificity of the model were evaluated by ROC curve, and the predictive ability of the prognostic risk model was determined by calculating the area under the curve (AUC). After constructing the 1-year, 3-years, and 5-years ROC charts, the 1-year ROC chart had the largest AUC score was found, and Akaike Information Criterion (AIC) scores for each point of the 1-year ROC chart to detect the maximum inflection point were calculated. This value was selected as the critical value to distinguish between high-risk and low-risk ccRCC patients. CcRCC patients were ranked according to risk scores, which were visualized as the distribution of risk scores, and assessed the number of patients in the high-risk and low-risk segments by distribution curves and scatter plots. Kaplan-Meier curve was used to analyze the survival prognosis of high-risk sites and low-risk sites.

### Correlation analysis of clinicopathological features and analysis of independent prognostic factors

To validate the clinical utility of constructed marker, we analyzed the relationship among risk scores as well as clinicopathological features (survival status, age, gender, tumor grade, clinical stage, and T, M, and N stage) with a chi-square test. Wilcoxon signed-rank test was used to study distinctions of these clinic pathological characteristics among high-risk as well as low-risk parts, and box plots were applied to display the results of the analysis. To evaluate the precision of the prognosis model in the aspects of prognosis living results, univariate and multivariate Cox regression analyses were performed on risk scores and clinicopathological features, identifying independent risk factors and displaying the results using forest maps. At the same time, in order to compare the accuracy of the risk score and clinical characteristics in predicting life, we plotted the risk score and clinicopathological characteristics in the same graph and drew the ROC curve of 1 year for comparison.

### Tumor-infiltrating immune cells analysis

To investigate the relationship between this marker and tumor-infiltrating immune cells, we first counted penetration values of the ccRCC dataset specimen based on seven currently recognized algorithms: XCELL (Aran et al., 2017), TIMER (Li et al., 2020), QUANTISEQ (Finotello et al., 2019), MCPCOUNTER (Dienstmann et al., 2019), EPIC (Racle and Gfeller, 2020), CIBERSORT-ABS (Tamminga et al., 2020), and CIBERSORT (Newman et al., 2015). Spearman Correlation study was used to evaluate the relationship between risk Score value and tumor-infiltrating immune cells. The results were shown in the bubble chart, based on *p* < 0.05 The process is performed with the R ggplot2 package.

### Immune checkpoints correlation analysis

To investigate whether there is differential expression of immune checkpoint-related genes in high and low-risk patients. We plotted expressions of CTLA4, GAL9, LAG3, PD-1, PD-L1, PD-L2, TIGIT, and TIM-3, separately, contrasted the difference using the Wilcoxon signed-rank test, and visualized them using the ggstatsplot package as well as violin plots.

### Targeted drug sensitivity analyses

The half-inhibition rate (IC50) of the drug was used as the index of drug sensitivity. And in order to evaluate the clinical signature for ccRCC treatment, we counted IC50 of commonly used targeted medicines for ccRCC, including axitinib, bevacizumab, pazopanib, sorafenib, and sunitinib. IC50 differences between high-risk as well as low-risk parts were contrasted *via* Wilcoxon signed-rank test, and consequences could be visualized with pRRophetic and ggplot2 packages in R software.

### Statistical analyses

Statistical analysis was performed in R software (version 4.1.1). The prognostic significance was assessed by univariate, Lasso, and multivariate Cox regression analysis. Kaplan-Meier survival curve study was used to analyze OS. ROC profile analysis and its AUC values were applied to evaluate the reliability and sensitivity of prognostic signature. Spearman correlation test was used for risk score correlation analysis. *p* < 0.05 was considered as a significant difference among all tests.

## Results

### Identification of nrlncRNAs in ccRCC Patients

**FIGURE 2:**
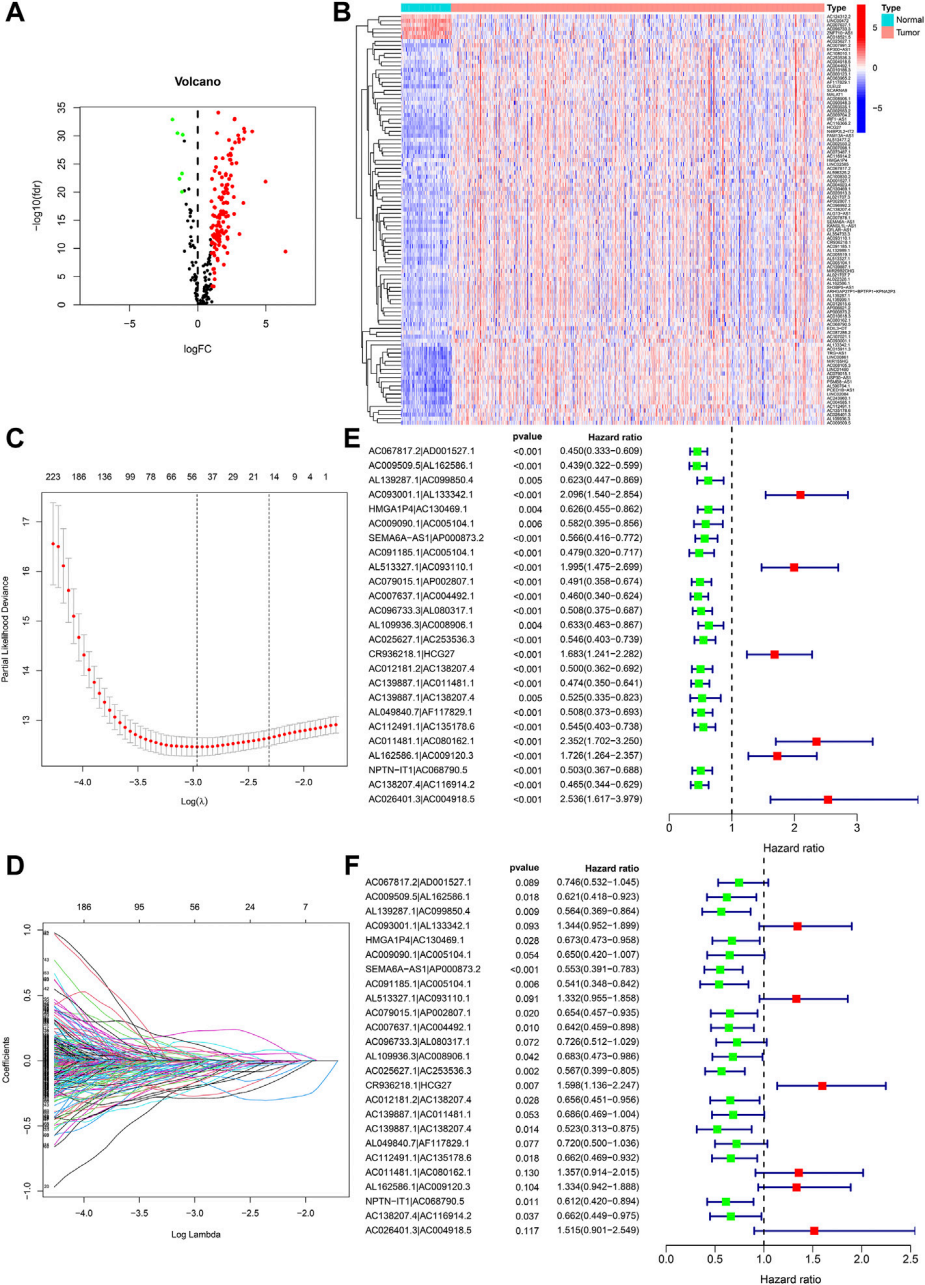
Development of nrlncRNA pairs prognostic signature. **(A)** Heat maps of 136 DEnrlncRNAs. **(B)** Volcano map of 136 DEnrlncRNAs. **(C)** Establishment of prognostic DEnrlncRNAs LASSO regression. **(D)** Distribution of LASSO coefficients for 50 DEnrlncRNA pairs. **(E)** Univariate Cox hazard analyses were conducted for 25 DEnrlncRNA pairs. **(F)** Multivariate Cox hazard analyses were conducted for 25 DEnrlncRNA pairs.

The detailed process of our study is illustrated in Figure 1. Transcriptome analysis information and clinical information of ccRCC were obtained from the TCGA database, which contained 539 ccRCC tissue specimens as well as 72 ordinary kidney tissue specimens. We found that 533 specimens with fully clinical information could be selected for follow-up analysis. The information could be noted according to a human Gene Transfer Format (GTF) annotation file, 67 necroptosis-related genes were obtained according to previous studies (Supplementary Table S2 **)**, and a Pearson correlation study was used to verify 347 nrlncRNAs (Supplementary Table S3) with correlation coefficients >0.6 and *p* < 0.001. Further differences show the study was performed using |log fold change| >1.0 and false discovery rate (FDR) <0.05 as screening standards. We obtained 136 DEnrlncRNAs, 6 down-regulation lncRNAs and 130 up-regulation lncRNAs were included (Figure 2A; Supplementary Table S4), and gene heatmaps were generated using R software (Figure 2B).

### Construction of a prognosis signature according to DEnrlncRNA pairs

A total of 5,434 DEnrlncRNA pairs were screened from 136 DEnrlncRNAs by using an iterative cycle method as well as a 0 or one matrix (Supplementary Table S5). We screened out 50 DEnrlncRNA pairs with a univariate test and LASSO regression study (Figures 2C,D), followed by a multivariate Cox regression study, of which 25 were included in the prognostic signature based on a stepwise approach (Figure 2F; Table 1). Figure 2E shows the univariate Cox regression analysis results of lncRNA.

**TABLE 1:** 25 prognostic nrlncRNA pairs multivariate COX regression study results.

LncRNAs	Coefficient	HR	HR.95L	HR.95H	*p*-value
AC067817.2|AD001527.1	−0.2929	0.7461	0.5325	1.0455	0.0888
AC009509.5|AL162586.1	−0.47579	0.6214	0.4184	0.9231	0.0184
AL139287.1|AC099850.4	−0.57189	0.5645	0.3687	0.8643	0.0085
AC093001.1|AL133342.1	0.2959	1.3443	0.9518	1.8987	0.0930
HMGA1P4|AC130469.1	−0.3960	0.6730	0.4728	0.9580	0.0279
AC009090.1|AC005104.1	−0.4302	0.6504	0.4200	1.0071	0.0538
SEMA6A-AS1|AP000873.2	−0.5920	0.5532	0.3909	0.7829	0.0008
AC091185.1|AC005104.1	−0.6144	0.5410	0.3477	0.8415	0.0064
AL513327.1|AC093110.1	0.2868	1.3322	0.9552	1.8580	0.0910
AC079015.1|AP002807.1	−0.4251	0.6537	0.4571	0.9349	0.0199
AC007637.1|AC004492.1	−0.4435	0.6418	0.4586	0.8982	0.0097
AC096733.3|AL080317.1	−0.3207	0.7256	0.5115	1.0293	0.0722
AL109936.3|AC008906.1	−0.3817	0.6827	0.4728	0.9858	0.0417
AC025627.1|AC253536.3	−0.5682	0.5665	0.3988	0.8049	0.0015
CR936218.1|HCG2; 7	0.4685	1.5976	1.1359	2.2469	0.0071
AC012181.2|AC138207.4	−0.4211	0.6563	0.4508	0.9556	0.0281
AC139887.1|AC011481.1	−0.3766	0.6862	0.4688	1.0044	0.0527
AC139887.1|AC138207.4	−0.6484	0.5229	0.3126	0.8747	0.0135
AL049840.7|AF117829.1	−0.3290	0.7196	0.4997	1.0362	0.0770
AC112491.1|AC135178.6	−0.4132	0.6615	0.4693	0.9324	0.0183
AC011481.1|AC080162.1	0.3053	1.3570	0.9140	2.0150	0.1301
AL162586.1|AC009120.3	0.2880	1.3338	0.9421	1.8882	0.1044
NPTN-IT1|AC068790.5	−0.4904	0.6124	0.4195	0.8939	0.0110
AC138207.4|AC116914.2	−0.4130	0.6617	0.4490	0.9752	0.0369
AC026401.3|AC004918.5	0.4155	1.5152	0.9007	2.5490	0.1174

Abbreviations: HR, hazard ratio; HR.95L: 95% CI, lower limit; HR.95H: 95% CI, upper limit.

### Validation of prognostic signature based on DEnrlncRNA pairs

**FIGURE 3:**
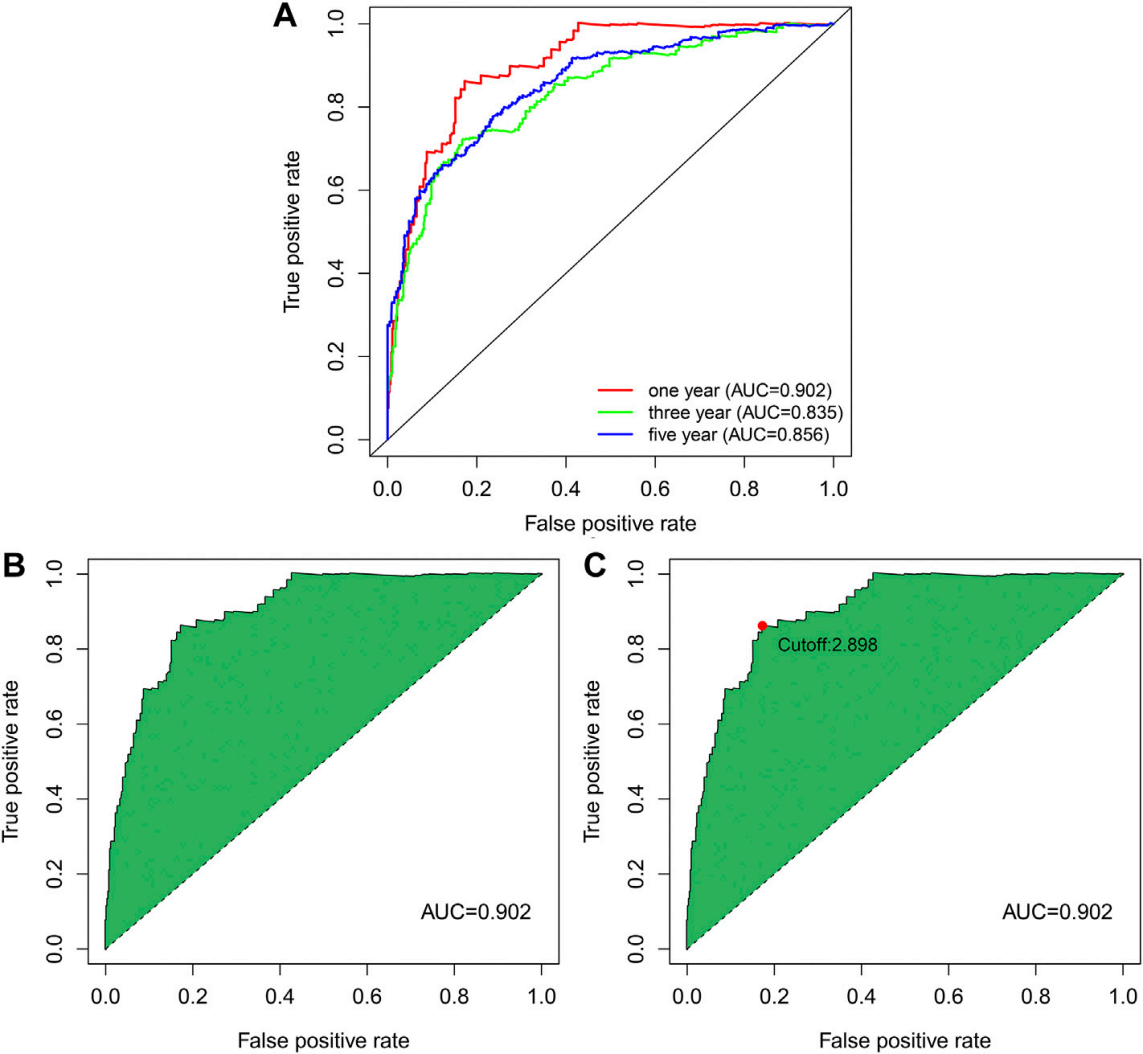
ROC curve of nrlncRNA pairs signature. **(A)** 1-year, 3-years, and 5-years ROC profiles. **(B)** ROC curve with best AUC value over 1 year. **(C)** Cut-off point calculated by AIC.

ROC curves of the above 25 pairs of DEnrlncRNA subjects at 1-, 3-, and 5 years were plotted and AUC scores were collected. All AUC scores exceeded 0.8 (Figure 3A), and the maximum AUC at 1 year was 0.902 (Figure 3B). An AIC score with a cut-off point of 2.898 was found in the 1-year ROC curve. (Figure 3C).

### Clinical assessment by prognostic signature based on DEnrlncRNA pairs

**FIGURE 4:**
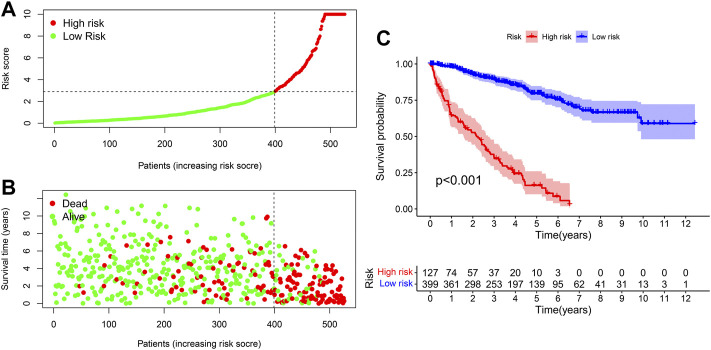
The signature for prognosis prediction in ccRCC. **(A)** Risk score curves in ccRCC patients. **(B)** Distribution of living conditions in patients with ccRCC. **(C)** Kaplan-Meier whole living profiles of high-risk as well as low-risk groups in prognostic signature.

**FIGURE 5:**
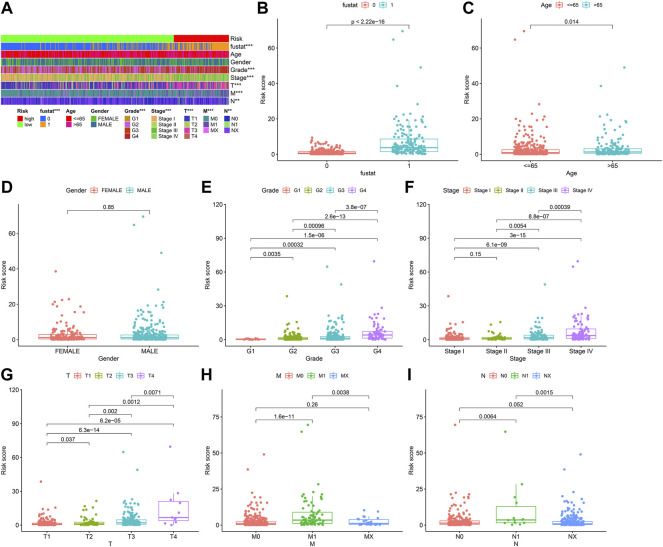
Clinical correlation of prognosis marker using clinic pathological features of ccRCC patients. **(A)** Bar charts summarizing common clinical features **(B)** Survival status (*p* < 0.001). **(C)** Age (*p* > 0.05). **(D)** Gender (*p* > 0.05). **(E)** Tumor grade (*p* < 0.001). **(F)** Clinical stage (*p* < 0.001). **(G)** T stage (*p* < 0.001). **(H)** M stage (*p* < 0.001). **(I)** N stage (*p* < 0.001).

We used the prognosis indicator model to calculate the risk score of all ccRCC patients. Patients were then divided into high-risk (*n* = 127) as well as low-risk (*n* = 399) (Figure 4A) according to the cutoff point calculated above. The survival status of the two cohorts of patients are shown in Figure 4B. According to the Kaplan-Meier test results, the clinical results of patients with low risk were better than those with high risk. The survival time of low-risk patients was longer than those at high risk (*p* < 0.001) (Figure 4C). We then used the Wilcoxon sign-rank test (Figure 5A) to create a heat map of the relationship between risk scores and clinical indicators. Then, we obtained a heatmap by Wilcoxon signed-rank test (Figure 5A) depicting the relation between risk scores as well as clinical indicators. Survival status (Figure 5B), tumor grade (Figure 5E), clinical stage (Figure 5F), T stage (Figure 5G), and M stage (Figure 5H), as well as N stage (Figure 5I) were remarkably correlated with risk score (*p* < 0.001), while age (Figure 5C) and gender (Figure 5D) were not remarkably correlated with risk score (*p* > 0.05).

### This prognostic signature as an independent prognostic predictor

**FIGURE 6:**
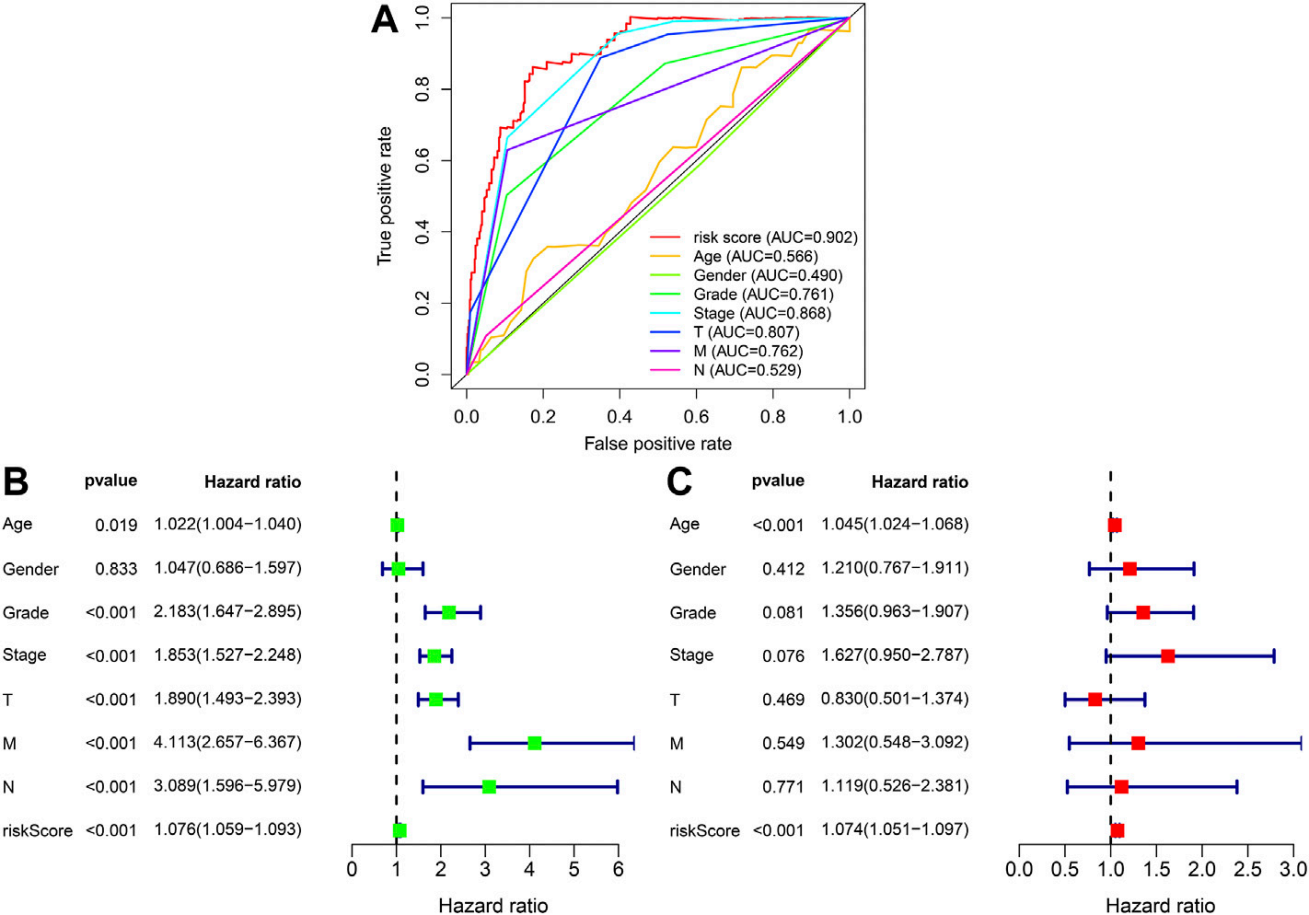
To assess forecasted independence of the prognostic marker for ccRCC prognosis. **(A)** 1-year ROC profile shows a higher risk score compared to other common clinicopathological features. **(B)** Univariate Cox regression analysis of risk score and clinicopathological characters. **(C)** Multivariate Cox regression analysis of risk score as well as clinic pathological characters.

To further explore the prognosis score of constructed prognostic marker in ccRCC, univariate as well as multivariate Cox regression analysis were conducted on risk scores and clinical indicators, and forest plots were drawn (Figure 6B and Figure 6C). Univariate Cox regression study indicated that age (*p* = 0.019, HR = 1.022, 95% CI [1.004–1.040]), grade (*p* < 0.001, HR = 2.183, 95% CI [1.647–2.895]), clinical stage (*p* < 0.001, HR = 1.853, 95% CI [1.527–2.248]), T stage (*p* < 0.001, HR = 1.890, 95% CI [1.493–2.393]), M Stage (*p* < 0.001, HR = 4.113, 95% CI [2.657–6.367]), N Stage (*p* < 0.001, HR = 3.089, 95% CI [1.596–5.979]), and risk score (*p* < 0.001, HR = 1.076, 95% CI [1.059–1.093]) were related to the overall survival, Multivariate Cox regression study indicated risk score (*p* < 0.001, HR = 1.074, 95% CI [1.051–1.097]) was related to overall survival and was an independent predictor of ccRCC. The ROC profiles of clinical indicators and risk scores were drawn to compare their 1-year survival prediction performance (Figure 6A). The results showed that the AUC score of the patients was the highest (0.902), which proved that the risk score had the best predictive ability.

### Correlation analysis between prognostic signature and tumor-infiltrating immune cells

**FIGURE 7:**
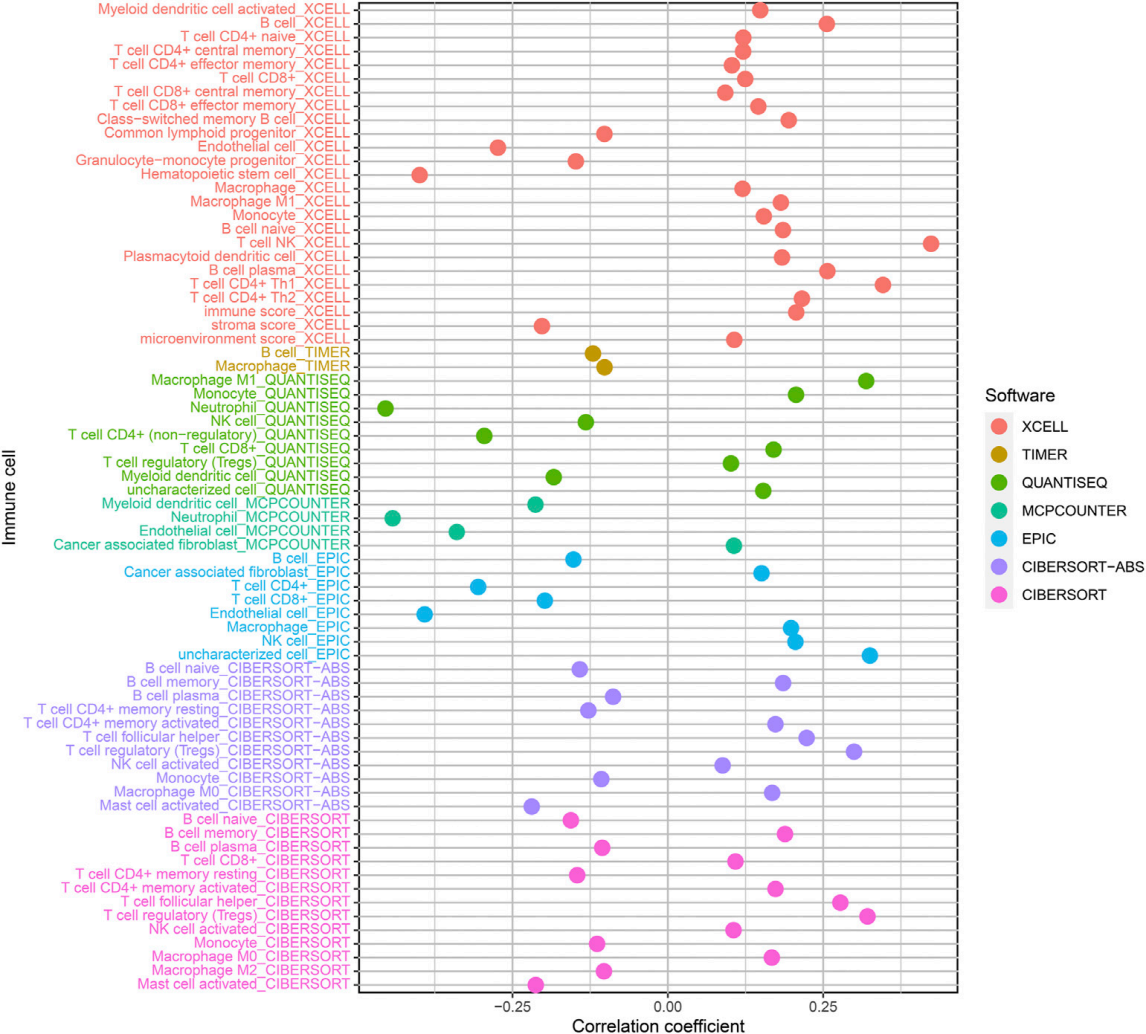
Correlation of tumor-infiltration immunity cells with the prognostic signature based on 7 known algorithms.

We used the Pearson correlation test to study the relationship between prognostic signature as well as tumor immunity infiltration cells based on seven algorithms, and the result presented a lollipop form, as indicated in Figure 7. Differences in tumor-infiltrating immune cells among high-risk and lowrisk cohorts are shown with boxplots (Supplementary Figure S1). Consequences indicated most immunity-infiltration cells in the ccRCC tumor micro circumstance (Supplementary Table S6) were negatively associated with high risk scores containing Granulocyte-monocyte progenitor, Hematopoietic stem cell, Macrophage M2, Myeloid dendritic cell, Neutrophil, T cell CD4^+^ memory resting, and T cell CD4^+^. Cells positively associated with high risk scores included B cell memory, cancer-related fibroblast, class-changed memory B cell, macrophage M0, macrophage M1, NK cell activation, T cell CD4^+^ central memory, T cell CD4^+^ memory activation, T cell regulatory (Tregs), and T cell CD8^+^ central memory.

### Correlation study between prognosis marker and immune checkpoint inhibitors

**FIGURE 8:**
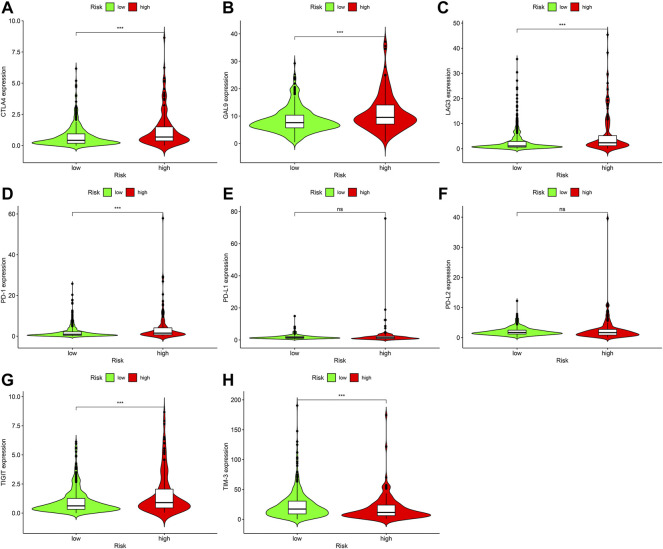
Relationship among prognostic signature as well as express standards of immunity checkpoint-associated genes, **(A)** CTLA4 (*p* < 0.001), **(B)** GAL9 (*p* < 0.001), **(C)** LAG3 (*p* < 0.001), **(D)** PD-1 (*p* < 0.001), **(E)** PD-L1 (*p* > 0.05), **(F)** PD-L2 (*p* > 0.05), **(G)** TIGIT (*p* < 0.001), **(H)** TIM-3 (*p* < 0.001).

Immune Checkpoint Inhibitors are one of the important treatments for ccRCC. We further discovered relationship between prognosis marker and immune checkpoint-related genes, and discovered that high risk scores were significantly associated with high express of CTLA4 (*p* < 0.001; Figure 8A), GAL9 (*p* < 0.001; Figure 8B), and LAG3 (*p* < 0.001; Figure 8C), PD-1 (*p* < 0.001; Figure 8D), and TIGIT (*p* < 0.001; Figure 8G) were positively correlated, while high risk score were negatively related to high express of TIM-3 (*p* < 0.001; Figure 8H), but PD-L1 (*p* > 0.05; Figure 8E) and PD-L2 (*p* > 0.05; Figure 8F) were not remarkably different from risk scores.

### Correlation analysis between prognostic signature and targeted drug sensitivity

**FIGURE 9:**
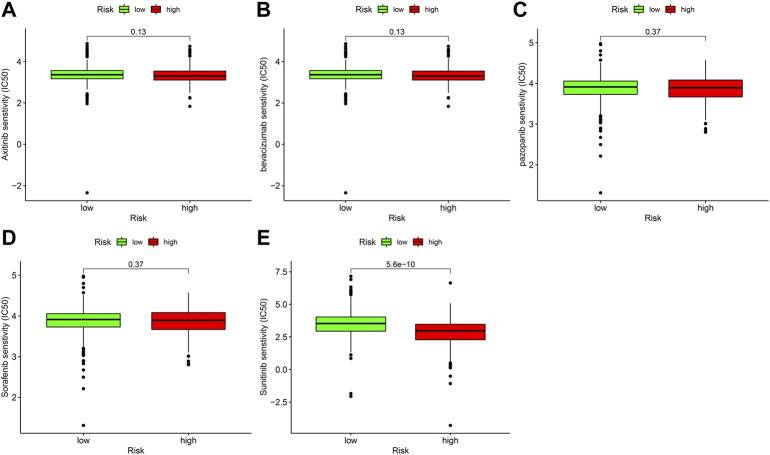
The relationship between prognostic signature and sensitivity of targeted drug. **(A)** axitinib (*p* > 0.05), **(B)** bevacizumab (>0.05), **(C)** pazopanib (>0.05), **(D)** sorafenib (>0.05), **(E)** sunitinib (*p* < 0.001).

Targeted medicines are the most important principal treatment for terminal ccRCC. We discovered the high-risk score was related to a low IC50 for sunitinib (Figure 9E), suggesting that this prognostic signature could be a potential prediction of sunitinib sensitivity. In contrast, the IC50 of axitinib (Figure 9A), bevacizumab (Figure 9B), pazopanib (Figure 9C), and sorafenib (Figure 9D) did not differ significantly between high-risk and low-risk sites.

## Discussion

CcRCC is a common urological malignancy and is the most universal kind of kidney cancer, comprising a proportion of 80% of total kidney cancer types, but more than 30% of sick persons with ccRCC have metastases at diagnosis (Zheng and Yang, 2017; Miller et al., 2019). Surgery is the primary therapy for early-stage clear cell carcinoma, however, molecularly targeted medicines are the primary treatment for advanced transferred clear cell carcinoma, which can significantly prolong the overall survival and non-developmental survival of ccRCC patients. But lack of markers of drug sensitivity to molecularly targeted drugs and the lack of molecule signatures of ccRCC metastasis have generated great difficulty for clinical therapy (Wettersten et al., 2017). Thus, it is quite vital important to discover susceptive and peculiar tumor signatures for clinical treatment of ccRCC patients and to make the survival rate of ccRCC patients better. In the paper, we obtained 50 differentially expressed nrlncRNA pairs and identified 25 nrlncRNA pairs significantly associated with ccRCC prognosis by univariate, LASSO, and multivariate Cox regression study to establish the signature of ccRCC based on necroptosis-associated lncRNA. Among these lncRNAs, studies reported that AL162586.1 was significantly associated with bladder cancer prognosis (Chen et al., 2021b). AL139287.1 was confirmed to be related to Head and Neck Squamous Cell Carcinoma prognosis (Shen et al., 2021). HMGA1P4 is highly expressed in gastric cancer tissues and promotes cisplatin resistance in gastric cancer (Qiao et al., 2020). Studies have reported that the low expression of SEMA6A-AS1 in hepatocellular carcinoma is associated with the poor prognosis of HBV-associated hepatocellular carcinoma (Yu et al., 2020). AC091185.1 has been published to be related to prognostic among patients with lung adenocarcinoma (Zheng et al., 2021a). AC093110.1 can adjust the expression of SPTBN1 in breast tumors as well as promote the proliferation and migration of breast cancer cells (Zhang et al., 2022). AP002807.1 was associated with prognosis in ccRCC (Li et al., 2021). The study reported that AC008906.1 forecast prognostic of acute myeloid leukemia (Zheng et al., 2021b). Notably, exploration of these newly discovered nrlncRNAs could lead to a better understanding of ccRCC and perhaps new goals of ccRCC therapy. Then, according to the AIC optimal fit to acquire critical value for differentiating between high as well as low-risk cohorts, patients with ccRCC were separated into a high-risk cohort and a low riskcohort. The results showed prognosis of patients in the low-risk group was remarkably better than that in the high-risk group patients. The risk score was an individual prediction of prognostic in patients with ccRCC. Meanwhile, the ROC curve verification indicated this marker was significantly better than the clinicopathological features in forecasting prognostic of ccRCC patients. In conclusion, these studies suggest that nrlncRNA marker can precisely forecast prognostic of ccRCC patients.

Tumor-infiltration immune cells are an important part of the tumor immunity micro circumstance and exert a vital regulatory part in tumor development and metastasis (Grivennikov et al., 2010). To discover the relationship between risk scores and tumor-infiltration immune cells, we applied seven approaches for evaluating tumor-infiltration immunity cells as well as showed that B cell memory, cancer-related fibroblast, class-changed memory B cell, macrophage M0, macrophage M1, NK cell activation, T cell CD4^+^ central memory, T cell CD4^+^ memory activation T cell regulatory (Tregs), and T cell CD8^+^ central memory was positively associated with risk scores. Previous studies have shown that ccRCC is one of the most immune-infiltration cancers, with high levels of CD8^+^ T cell, B cell memory, and T cell regulated (Tregs) infiltration. It is related to poor prognostic in ccRCC (Vuong et al., 2019; Pan et al., 2020). Moreover, studies have confirmed that high levels of macrophage M1 infiltration are remarkably related to whole living as well as illness-free living in ccRCC (Xu et al., 2020). We also performed a correlation study of immune checkpoint-associated genes as well as targeted drugs with risk scores and found that CTLA4, GAL9, PD-1, and TIGIT were explored to be positively related to risk score. TIM-3, however, was negatively correlated with risk scores. These immune checkpoint-associated genes can serve as potential therapeutic targets. At the same time, studies have also confirmed that PD-1 is highly expressed in ccRCC, while TIM-3 is underexpressed (Chevrier et al., 2017). Immune checkpoint inhibitors that block PD-1 and CTLA4 have been indicated to be efficient in the therapy of ccRCC and can be used as standard therapy (Motzer et al., 2018). Sunitinib is a principal targeted treatment drug for patients with terminal ccRCC (Bedke et al., 2017). Our prognostic signature showed patients in the high-risk group were more sensitive to sunitinib than those in low-risk group. Identifying the sensitivity of patients with advanced ccRCC to sunitinib may reduce the cost of therapy and reduce drug side effects (Xie et al., 2021). But we are also aware of some limitations and deficiencies of this study. First, our research lacked external validation from other clinic datasets. Second, the potential molecule mechanism of nrlncRNA in ccRCC needs further verification through molecular experiments. Third, the specific mechanism between nrlncRNA on markers and immunotherapy effect in ccRCC patients was not clarified. Thus, in our next study, we will gather clinical specimens for further validation and further exploration through laboratory experiments, but its evaluation will take a long time. In summary, we established a new nrlncRNA pair marker in ccRCC patients in the paper, by integrating of 25 nrlncRNA pairs, which was a single predictor of prognosis in ccRCC patients, and has a subject operating characteristic region of 0.902, 0.835, and 0.856 at 1, 3, and 5 years, respectively. Cox regression and stratified survival study showed this marker could be an independent predictor of ccRCC patients. Furthermore, patients with different risk scores had significant differences in tumor-infiltrating immune cells, immune checkpoint, and semi-inhibitory concentration of targeted drugs, suggesting that this marker could be used to evaluate the clinical efficacy of immunotherapy and targeted drug therapy. This marker predicts immunotherapy efficacy and sunitinib sensitivity in ccRCC patients.

Studies have also shown that necroptosis-related genes are promising biomarkers for predicting prognosis and treatment response in ccRCC (Luo and Zhang, 2022). Necroptosis-related signatures have also been demonstrated as novel prognostic predictors of immune microenvironment and treatment response in ccRCC (Chen et al., 2022b). The results of this study also show that nrlncRNAs pair markers may help to evaluate the prognosis and molecular characteristics of ccRCC patients, as previously reported.

In conclusion, this study demonstrated that 25-nrlncRNAs pairs marker may contribute to evaluating prognostic and molecular characteristics of ccRCC patients, improve treatment methods, and can be more used in clinical practice.

## Data Availability

The original contributions presented in the study are included in the article Supplementary Material, further inquiries can be directed to the corresponding authors.
