# Supplementary figures and images for: A novel marker based on necroptosis-related long non-coding RNA for forecasting prognostic in patients with clear cell renal cell carcinoma
Source: Front Genet. 2022 Sep 21;13:948254. doi: 10.3389/fgene.2022.948254 (PMC9532702; doi:10.3389/fgene.2022.948254)

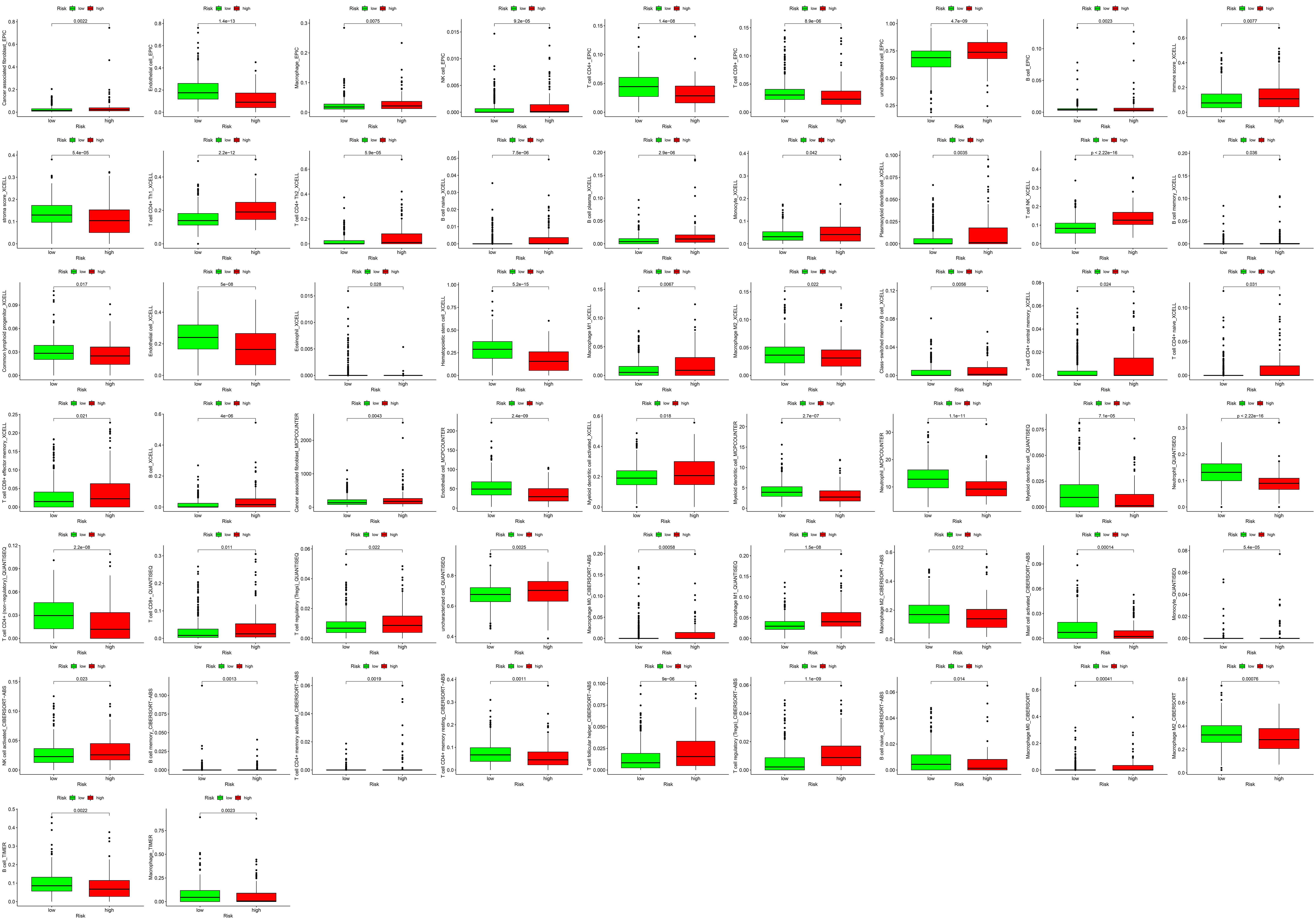

Supplement: Supplementary file 3 [file Image1.tif]
